# Clinical significance of T helper cell subsets in the peripheral blood and bone marrow of patients with multiple myeloma

**DOI:** 10.3389/fimmu.2024.1445530

**Published:** 2024-09-11

**Authors:** Liangjun Zhang, Huixiu Zhong, Jiwen Fan, Jiansen Mao, Yi Li

**Affiliations:** ^1^ Department of Laboratory Medicine, Zigong First People’s Hospital, Zigong, China; ^2^ Department of Laboratory Medicine, West China Hospital, Sichuan University, Chengdu, China; ^3^ Department of Laboratory Medicine, Nanjing International School, Nanjing, China

**Keywords:** multiple myeloma, T helper cells, T follicular helper, chemotherapy, prognosis

## Abstract

**Background:**

T helper (Th) cell subsets primarily assist B cells in differentiating into plasma cells in the germinal center. The mechanism of malignant transformation of plasma cells is an important target for the clinical treatment of MM; however, the mechanism remains unclear.

**Methods:**

We collected the peripheral blood (PB) and bone marrow (BM) samples of 33 patients with MM. In addition, the PB was also collected from 25 normal healthy controls (HCs). We analyzed the percentages of Th cell subsets in the PB and BM samples of patients with MM.

**Results:**

Tfh/CD4^+^ were positively correlated with the proportion of myeloma cells in the BM and PB samples (r = 0.592, P = 0.002 and r = 0.510, P = 0.010 respectively), and showed a strong correlation between the BM and PB samples (r = 0.6559, P = 0.0095). In the PB samples, the percentages of Th2/CD4^+^ and Tfh2/Tfh cells were significantly lower in patients with MM than in HCs (P = 0.00013 and P = 0.0004, respectively), whereas the percentage of Th17/CD4^+^ and Tfh17/Tfh was significantly higher in newly diagnosed patients with MM than in HCs (P = 0.0037 and P = 0.03, respectively), and all these cells showed a good predictive value for MM (area under the curve [AUC] 0.781, = 0.792, = 0.837, and 0.723 respectively). In the PB samples, all subsets of PD-1^+^ICOS^-^ Tfh showed a noticeable downward trend in MM from newly diagnosed to non-remission and remission groups. In contrast, all subsets of PD-1^-^ICOS^+^ Tfh increased gradually.

**Conclusion:**

Th cell subsets play an important role in the occurrence and development of MM and may provide a fundamental basis for identifying new immunotherapy targets and prognosis.

## 1 Introduction

Multiple myeloma (MM) is a type of blood cancer in which plasma cells (PCs) produce a large amount of monoclonal immunoglobulins that can be detected in the blood or urine of affected patients (1, 2). MM accounts for 10% of all hematologic malignancies (3). The incidence of MM has increased significantly in China from 2006 to 2018, with 20,066 new cases and 14,655 deaths, which was the highest worldwide (4). MM diagnosis requires ≥10% clonal bone marrow (BM) PCs or a biopsy-proven plasmacytoma with one or more multiple myeloma defining events: Hypercalcemia, renal failure, anemia, or lytic bone lesions attributable to the PC disorder, BM clonal plasmacytosis ≥60%, serum involved/uninvolved free light chain (FLC) ratio ≥100 (provided involved FLC is ≥100 mg/L), or >1 focal lesion on magnetic resonance imaging (5).

T helper cells (Th) express CD4 and CD3 and are classified as Th1, Th2, and Th17 cell subsets, which produce different cytokines and have different immunomodulatory functions. T follicular helper (Tfh) cells, a specialized subset of CD4^+^ T cells expressing chemokine (C-X-C motif) receptor 5 (CXCR5), programmed cell death 1 (PD-1), and inducible costimulatory molecule (ICOS), play a critical role in regulating the immune response by helping B cells produce high-affinity antibodies (6, 7). B cells that receive sufficient assistance from Tfh cells develop into long-lived plasma and memory B cells (8). Different isotypes of antibodies secreted by PCs have different functional capabilities (9). However, MM is characterized by malignant PC accumulation in the BM and the aberrant production of immunoglobulins (10). Therefore, as a key factor involved in B-cell maturation and differentiation, Tfh cells may play an important role in MM. A previous study reported that significantly increased Tfh cell ratios were found in patients with acute lymphoblastic leukemia (ALL), non-Hodgkin lymphoma, and MM (11). Tfh cells are classified as Tfh1, Tfh2, and Tfh17 based on CXCR3 and CCR6 expression (12). These Tfh subsets are associated with several diseases (13, 14). Notably, Tfh1, Tfh2, and Tfh17 cells have been detected in the peripheral blood (PB) of the patients with MM using flow cytometry, and a significantly increased Tfh17/Tfh ratio has been observed, especially in patients with relapsed MM (15), suggesting that Tfh17 might play a role in MM. However, data regarding the distribution of Tfh cell subsets in the BM is lacking.

To date, the complex relationship between Th cell subsets and MM is not yet fully understood. In this study, we analyzed the proportions of Th1, Th2, Th17, and Tfh subsets (Tfh1, Tfh2, and Tfh17) in the BM and PB to explore the relationship between Th subsets and the development and progression of MM.

## 2 Patients and methods

### 2.1 Patients

All samples were collected from inpatients of Zigong First People’s Hospital, the study protocol was approved by the Ethics Committee of the Zigong First People’s Hospital (NO.03202024). We enrolled 33 patients with MM, all patients received chemotherapy with bortezomib + lenalidomide + dexamethasone (including 15 newly diagnosed patients, 10 patients with incomplete remission after chemotherapy, and 8 patients with complete remission), who visited Zigong First People’s Hospital between 2021 and 2023. BM and PB samples were collected from the patients. The diagnosis and efficacy evaluation of patients with MM were based on the 2016 International Myeloma Working Group (IMMG) criteria (16). The exclusion criterion was a history of other hematological or autoimmune diseases. We enrolled 25 healthy controls (HCs) during routine health checkups and collected PB samples. The exclusion criteria were abnormal routine blood indicators, liver and kidney function indicators, history of genetic disease and obesity, recent illness, surgery, transfusion, and hospitalization. The demographic characteristics of MM and HCs showed **Table 1**.

**Table 1:** Demographic characteristics of MM patents and HCs.

Characteristics	MM (n = 33)	HC (n = 25)	P value	newly diagnose (n = 15)	incomplete remission (n = 10)	complete remission (n = 8)	P value
Age (years), mean (SD)	65.7 (10.2)	61.3 (10.4)	0.270	66.7 (9.83)	64.7 (12.7)	65.1 (8.5)	0.887
Male, n (%)	20 (60.6)	14 (56.0)	0.895	9 (60.0)	6 (60.0)	5 (62.5)	0.992
Anemia, n (%)	22 (66.7)	–	0.559	14 (93.3)	8 (80.0)	–	0.315
Renal insufficiency, n (%)	17 (51.5)	–	–	11 (73.3)	6 (6.00)	–	0.320
Bone disease, n (%)	10 (30.3)	–	–	7 (46.6)	3 (30.0)	–	0.405
Calcium elevation, n (%)	14 (42.4)	–	–	8 (53.3)	6 (60.0)	–	0.742

### 2.2 Flow cytometric analysis

The BM and PB samples with EDTA anticoagulant were diluted with phosphate-buffered saline (BC, California, USA) (PBS) at a ratio of 1:1. The Ficoll separation solution (BC, California, USA) was added to a centrifuge tube. A mixture of PB and PBS was slowly added along the wall of the centrifuge tube and centrifuged at 500 x g for 20 min to isolate PB mononuclear cells (PBMCs). The PBMC layer was carefully separated and transferred to a new centrifuge tube. Next, PBS solution was added to the tube, mixed thoroughly, centrifuged at 1500 rpm for 10 min, and washed twice after discarding the supernatant. The concentration of PBMCs was adjusted to 10^6^/mL, and 100 µL of PBMCs were added into two test tubes. CD4-APC750 (Beckman Coulter, REF: A94682, USA) CD3-ECD (Beckman Coulter, REF: A07748, USA), CXCR5-APC (BD Biosciences, Cat:558113, USA), CCR6-PC5.5 (BD Biosciences, Cat:566477, USA), CXCR3-PC7 (BD Biosciences, Cat:560831, USA), PD1-FITC (BD Biosciences, Cat:564494, USA), and ICOS-APC700 (BD Biosciences, Cat:566990, USA) antibodies were added to one test tube, and the other test tube added isotype control antibodies IgG2b-APC (BD Biosciences, Cat:557691, USA), IgG1-PC5.5 (BD Biosciences, Cat:566404, USA), IgG1-FITC (BD Biosciences, Cat:564416, USA), IgG1-APC700 (BD Biosciences, Cat:557873, USA), IgG1-PC7 (BD Biosciences, Cat:557872, USA). The solutions were mixed well and incubated at room temperature (20–25°C) in the dark for 15 min. Each tube was washed with 1 mL PBS and centrifuged at 500 x g for 5 min. Subsequently, the supernatant was discarded, and the cells were resuspended in 200 μL of PBS. Beckman Coulter NAVIOS flow cytometer (BC, California, USA) was used in this study. Data on 20,000 T cells were acquired on a NAVIOS instrument (BC) and analyzed using the Kaluza analysis software version 2.1 (BC). The control tube serves for distinguishing negative and positive cells. All experiments were performed in accordance with relevant guidelines and regulations.

**Figure 1:**
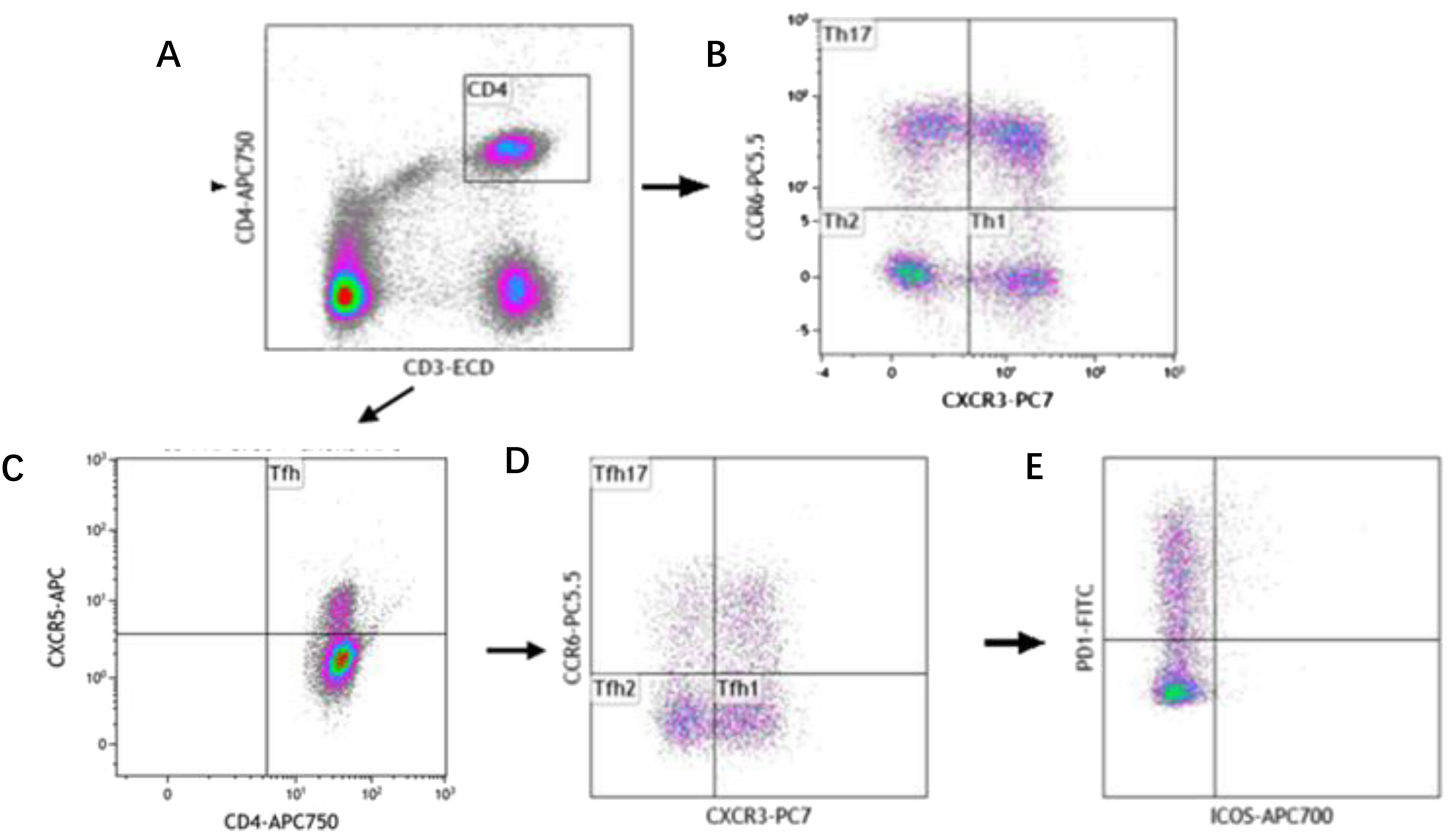
Reprehensive gating strategy for circulating Th cell subsets. **(A)** Schematic diagram of the flow cytometry analyses of CD3^+^ CD4^+^ Th cells. **(B)** Flow cytometry analyses of CXCR3^+^ CCR6^-^ Th1 cells, CXCR3^-^ CCR6^-^ Th2 cells, CXCR3^-^ CCR6^+^ Th17 cells. **(C)** Flow cytometry analyses of CD3^+^ CD4^+^ CXCR5^+^ Tfh cells. **(D)** Flow cytometry analyses of CXCR3^+^ CCR6^-^ Tfh1 cells, CXCR3^-^ CCR6^-^ Tfh2 cells, CXCR3^-^ CCR6^+^ Tfh17 cells. **(E)** The proportion of PD-1 and ICOS on Tfh cell subtypes.

The analysis of Th subset cells was carried out in the following steps successively, as shown in **Figure 1**. First, Th cells were circled through CD3^+^CD4^+^, and then, Th1, Th2 and Th17 cells were separated by cross gates using CXCR3 and CCR6. Tfh cells were circled by CD4^+^CXCR5^+^, and then CXCR3 and CCR6 were used to separate Tfh1, Tfh2 and Tfh17 cells with cross gates. Finally, PD-1 and ICOS cells were analyzed respectively within the gating of each Tfh subgroup.

The following cell subtypes were analyzed in this study: Th cells (CD4^+^CD3^+^), Th1 cells (CD3^+^CD4^+^CXCR3^+^CCR6^-^), Th2 cells (CD3^+^CD4^+^CXCR3^-^CCR6^-^), Th17 cells (CD3^+^CD4^+^CXCR3^-^CCR6^+^), Tfh cells (CD3^+^CD4^+^CXCR5^+^), Tfh1 cells (CD3^+^CD4^+^CXCR5^+^CXCR3^+^ CCR6^-^), Tfh2 cells (CD3^+^CD4^+^CXCR5^+^CXCR3^-^CCR6^-^), Tfh17 cells (CD3^+^CD4^+^CXCR5^+^CXCR3^-^CCR6^+^), PD-1^+^ICOS^-^ Tfh cells, PD-1^+^ICOS^-^ Tfh1 cells, PD-1^+^ICOS^-^ Tfh2 cells, PD-1^+^ICOS^-^ Tfh17 cells, PD-1^+^ICOS^+^ Tfh cells, PD-1^+^ICOS^+^ Tfh1 cells, PD-1^+^ICOS^+^ Tfh2 cells, PD-1^+^ICOS^+^ Tfh17 cells, PD-1^-^ICOS^+^ Tfh cells, PD-1^-^ICOS^+^ Tfh1 cells, PD-1^-^ICOS^+^ Tfh2 cells, and PD-1^-^ICOS^+^ Tfh17 cells (**Figure 1**).

### 2.3 Statistical analyses

**Figure 2:**
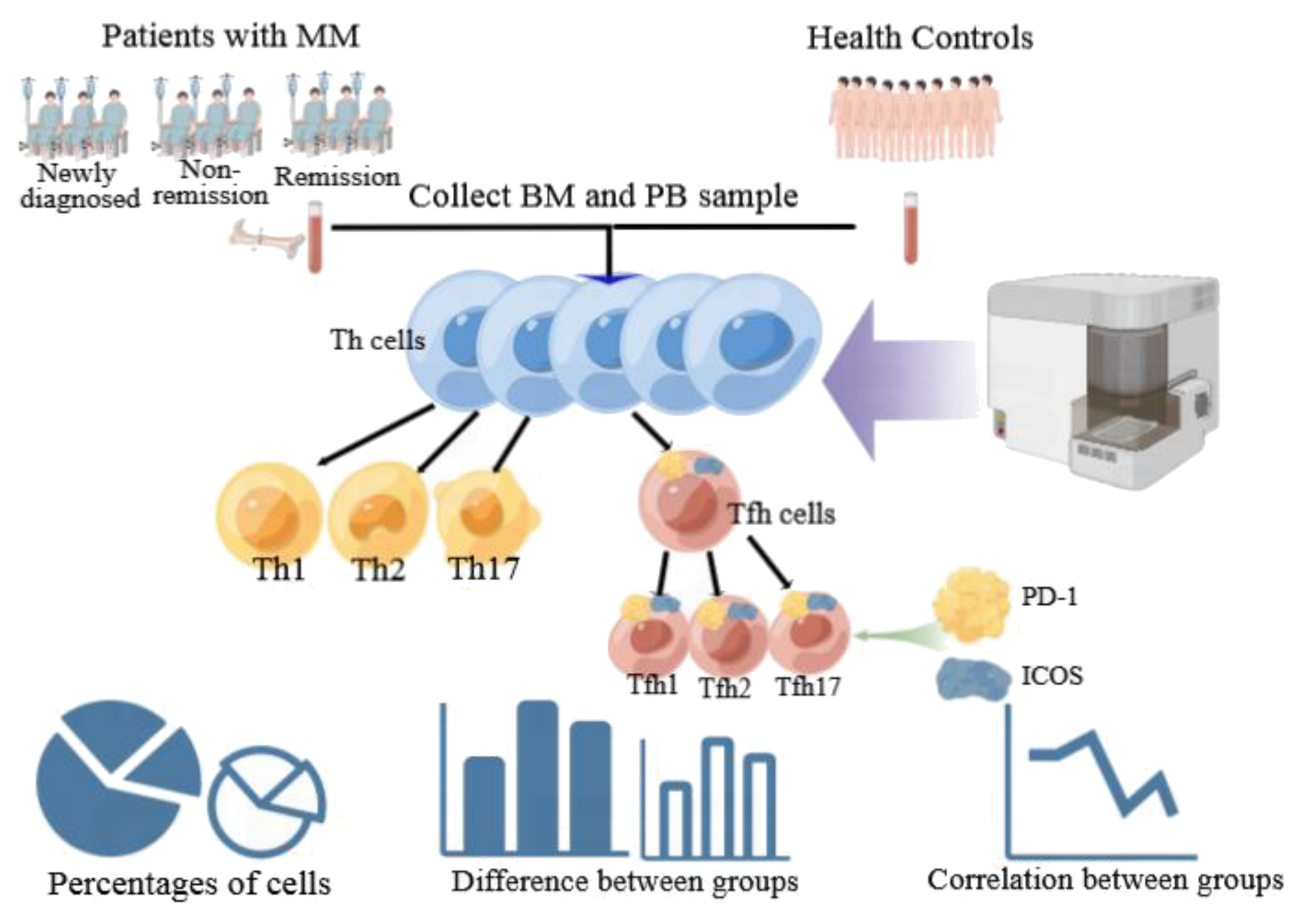
Technology roadmap of the study. This figure was drawn by Figdraw.

We used Kaluza software to summarize the flow cytometer data, and GraphPad Prism 5.01 software was used to further analyze and visualize the summarized data. Non-normal distribution was observed for all cell types, so the median and interquartile was used to describe the data for each group. For non-normally distributed lymphocyte subsets, comparisons between different two groups were performed using Mann–Whitney tests. The Kruskal–Wallis test was used for comparisons between multiple groups. Pearson’s correlation test was used for the correlation analysis. Receiver operating characteristic (ROC) curves were analyzed to determine the diagnostic value of the disease. Statistical significance was set at P < 0.05. The technical scheme of this study is illustrated in **Figure 2**.

## 3 Results

### 3.1 Percentage of the Th cell subsets in the PB of patients with MM and HCs

**Figure 3:**
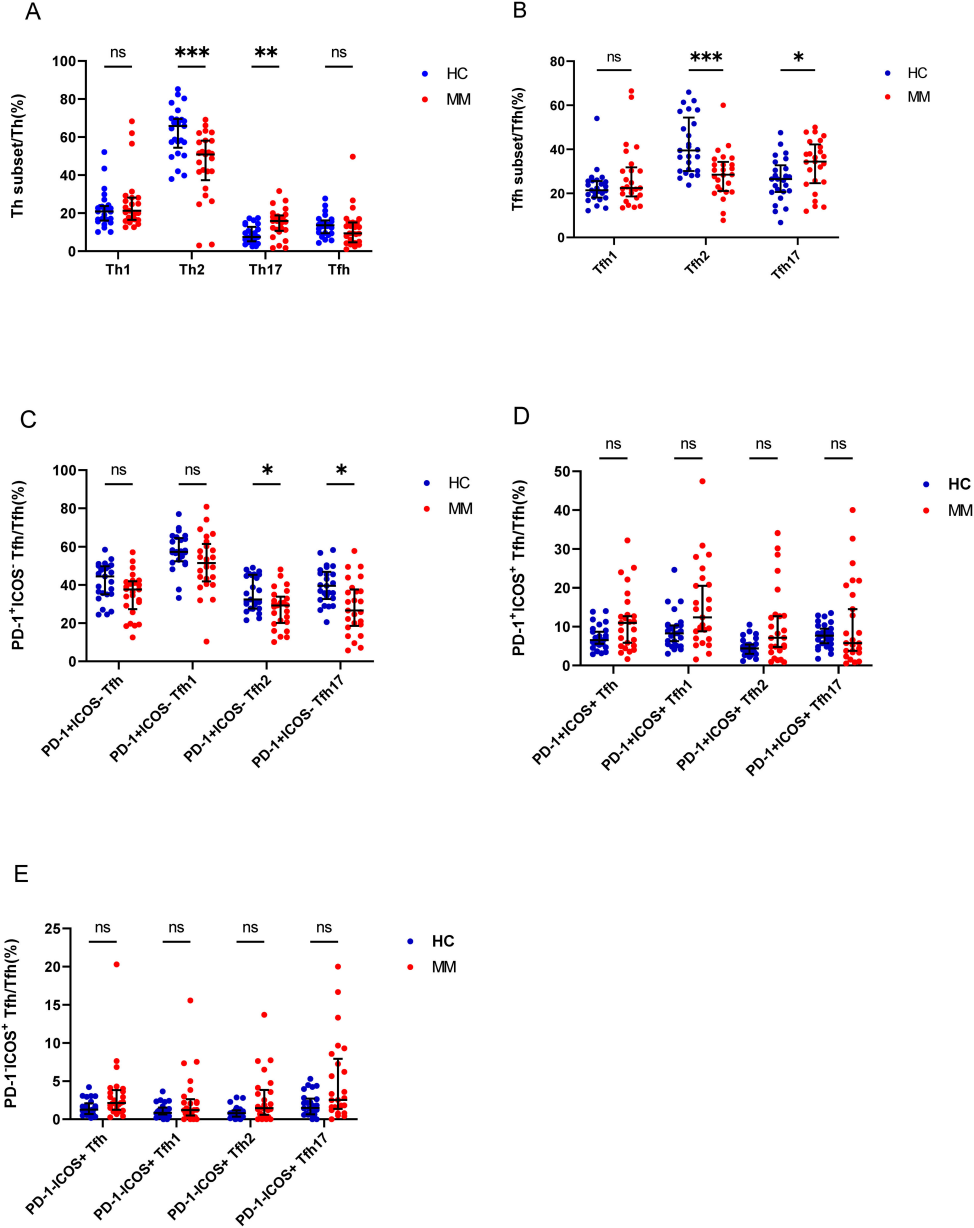
Th cell subsets in the PB samples of patients with MM compared to HCs. **(A)** Th subsets cell percentage among Th cells in patients with MM and HCs. **(B)** Tfh subsets cell percentage among Tfh cells in patients with MM and HCs. **(C)** PD-1^+^ICOS^-^ Tfh subsets cell percentage among Tfh cells in patients with MM and HCs. **(D)** PD-1^+^ICOS^+^ Tfh subsets cell percentage among Tfh cells in patients with MM and HCs.E. PD-1^-^ICOS^+^ Tfh subsets cell percentage among Tfh cells in patients with MM and HCs. “ns”, “*”, “**”, “***” represent not significant, P<0.05, P<0.01, P<0.001, respectively.

We analyzed the percentage of Th cell subsets in the PB samples of 33 patients with MM and 25 healthy controls. Notably, the percentage of Th2/CD4^+^ cells in patients with MM was significantly lower than that in HCs (P = 0.00013), but the percentage of Th17/CD4^+^ cells was significantly higher in patients with MM than that in HCs (P = 0.002) (**Figure 3A**). Among the Tfh subsets, the percentage of Tfh2/Tfh cells in patients with MM was significantly lower than that in HCs (P = 0.0004), in contrast, the percentage of Tfh17/Tfh cells was significantly higher in patients with MM than that in HCs (P = 0.03). However, no difference was observed in the percentages of Th1/CD4^+^ and Tfh1/Tfh cells between the two groups (P > 0.05) (**Figure 3B**).

Tfh cells were further classified as PD-1^+^ICOS^-^ Tfh cells, PD-1^+^ICOS^+^ Tfh cells, and PD-1^-^ICOS^+^ Tfh cells, and the percentages of PD-1^+^ICOS^-^ Tfh2 cells and PD-1^+^ICOS^-^ Tfh17 cells in patients with MM were lower than those in HCs (P = 0.01 and P = 0.003, respectively) (**Figure 3C**), but no differences were observed in the PD-1^+^ICOS^+^ Tfh cells between both groups (P > 0.05) (**Figure 3D**). In contrast, the percentages of PD-1^-^ICOS^+^ Tfh, PD-1^-^ICOS^+^ Tfh1, PD-1^-^ICOS^+^ Tfh2, and PD-1^+^ICOS^-^ Tfh17 cells in patients with MM were higher than those in HCs; however, the difference was not statistically significant (P > 0.05).

### 3.2 Percentage of Th subsets in the PB of patients with MM during different states

**Figure 4:**
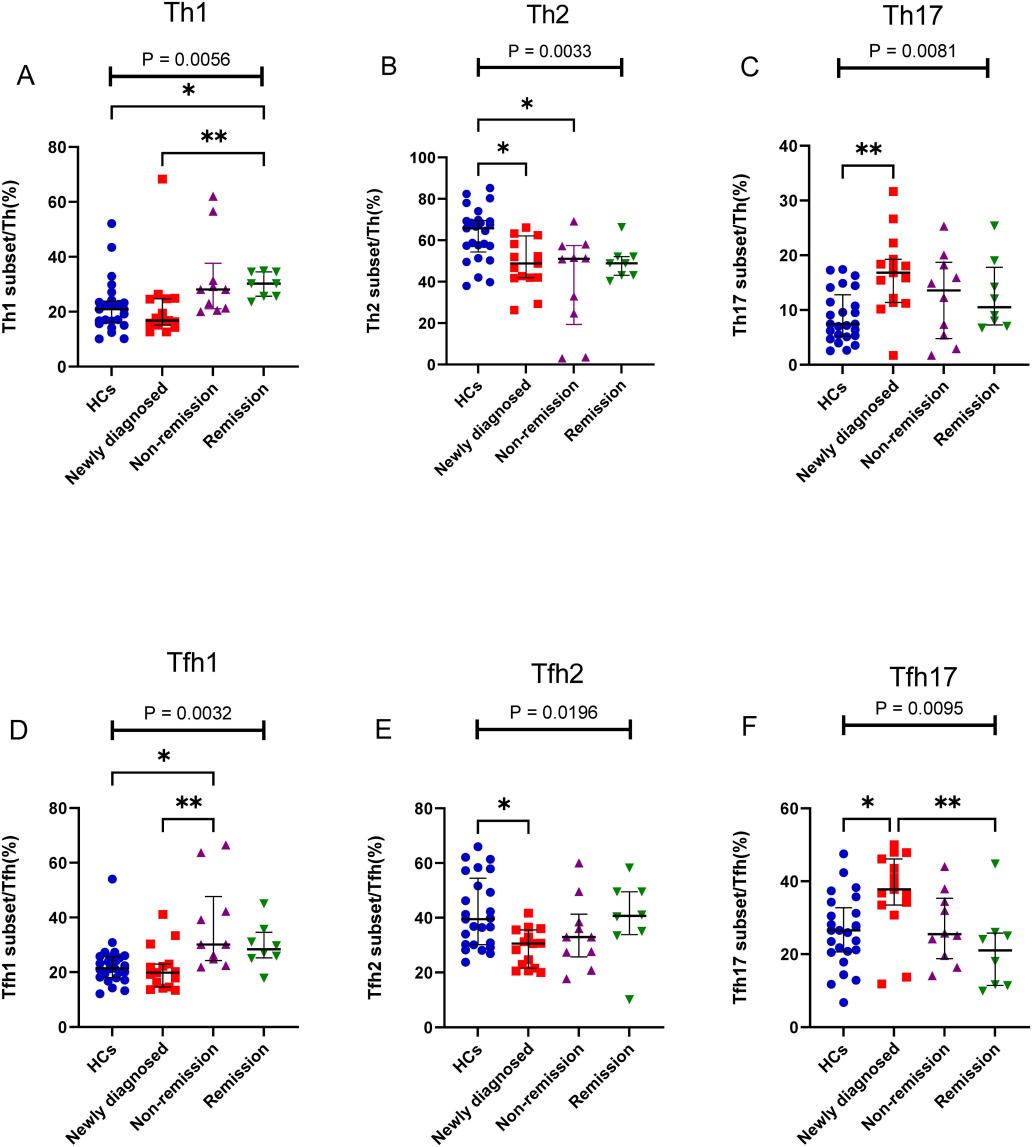
Th cell subsets in the PB samples of patients with MM during different states compared to HCs. **(A)** Th1 subsets cell percentage among Th cells in patients with MM during newly diagnosed, non-remission, and remission states. **(B)** Th2 subsets cell percentage among Th cells in patients with MM during newly diagnosed, non-remission, and remission states. **(C)** Th17 subsets cell percentage among Th cells in patients with MM during newly diagnosed, non-remission, and remission states. **(D)** Tfh1 subsets cell percentage among Tfh cells in patients with MM during newly diagnosed, non-remission, and remission states. **(E)** Tfh2 subsets cell percentage among Tfh cells in patients with MM during newly diagnosed, non-remission, and remission states. **(F)** Tfh17 subsets cell percentage among Tfh cells in patients with MM during newly diagnosed, non-remission, and remission states. “*”, “**” represent P<0.05, P<0.01, respectively.

Depending on the patient’s outcome after chemotherapy, we further divided the patients with MM into three groups (newly diagnosed, non-remission, and remission groups) and added HCs, it found significant differences between the four groups in Th1, Th2, and Th17 cells (P = 0.0056, P = 0.0033, and P = 0.0081, respectively) (**Figures 4A–C**). No difference was observed in the percentage of Th1 cells between the newly diagnosed groups and HCs (P > 0.05); however, after chemotherapy, the percentage of Th1/CD4^+^ cells gradually increased. Notably, the percentage of Th1/CD4^+^ cells was significantly higher in the remission group than that in the newly diagnosed groups (P = 0.0481) (**Figure 4A**). The percentage of Th2/CD4^+^ cells in newly diagnosed and non-remission patients was significantly lower than that in HCs (P = 0.0257 and P = 0.0162, respectively). However, after complete remission, the percentage of Th2/CD4^+^ cells did not differ between the remission group and HCs (P > 0.05) (**Figure 4B**). The percentage of Th17/CD4^+^ cells in patients with newly diagnosed MM was significantly higher than that in HCs (P = 0.0037), and after chemotherapy, a gradual downward trend was observed, and cells returned to approximately normal levels in the remission group (**Figure 4C**). No significant difference was observed in the percentage of Tfh cells among the four groups (P > 0.05). However, significant differences between the four groups were observed for Tfh1, Tfh2, and Tfh17 cells (P = 0.0032, P = 0.0054, and P = 0.0095, respectively). The percentage of the Tfh1 cells was similar to that of the Th1 cells, and no difference in their percentages was observed between the newly diagnosed group and HCs (P > 0.05). However, after chemotherapy, the percentage of Tfh1/Tfh cells in the non-remission group significantly increased compared to that in the newly diagnosed group and HCs (P = 0.007 and P = 0.02, respectively) (**Figure 4D**). The percentage of Tfh2/Tfh cells in the newly diagnosed group was lower than that in HCs (P = 0.02); however, it gradually increased from the newly diagnosed group to the remission group after chemotherapy. (**Figure 4E**). In contrast, the percentage of Tfh17/Tfh cells in newly diagnosed patients with MM was higher than that in HCs (P = 0.03); it returned to normal in the remission group after chemotherapy. Notably, difference in the percentage of Tfh17/Tfh cells among the four groups was observed (P = 0.0095) (**Figure 4F**).

**Figure 5:**
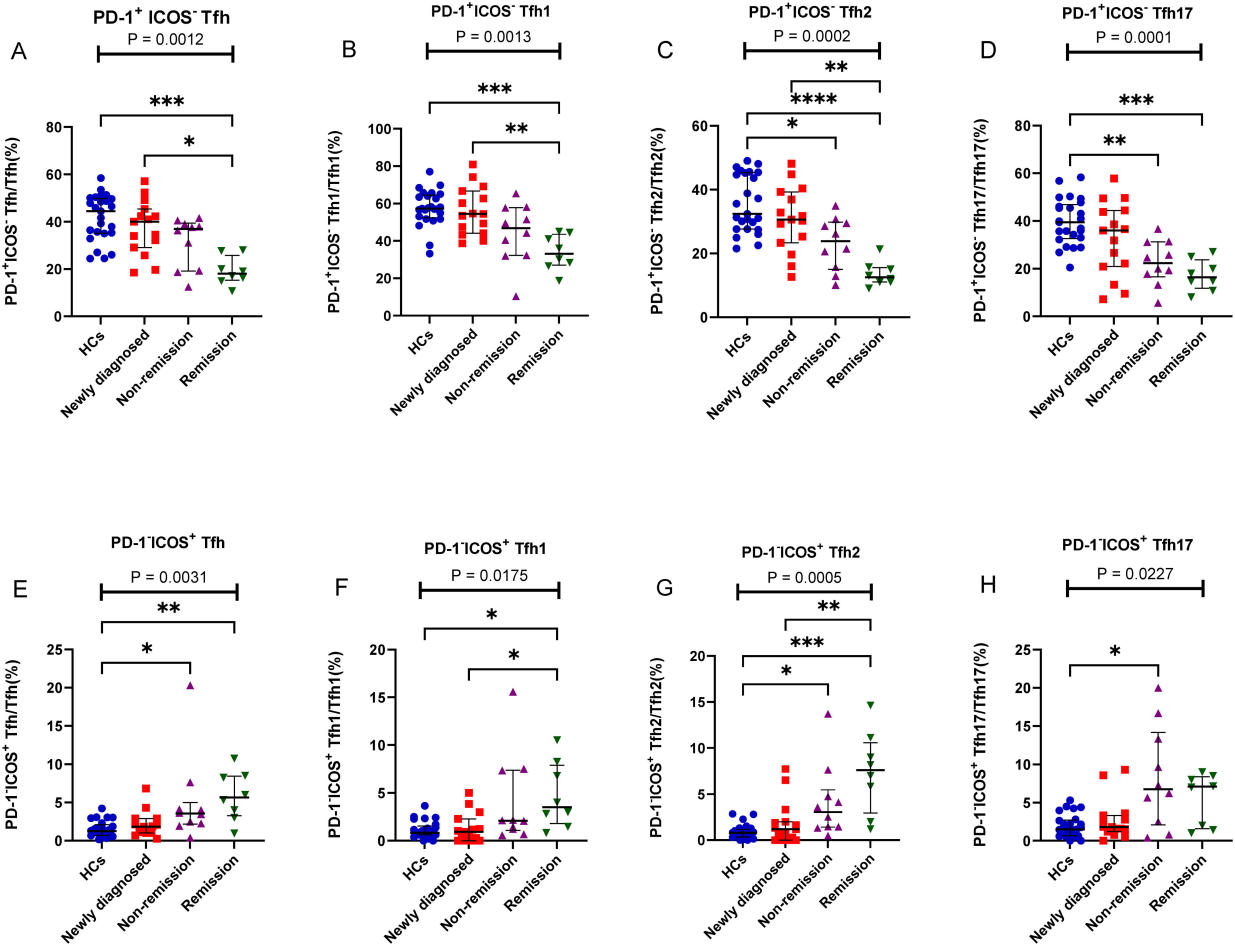
PD-1^+^ICOS^-^ Tfh and PD-1^-^ICOS^+^ Tfh cell subsets in patients with MM during different states compared to HCs. **(A)** PD-1^+^ICOS^-^ Tfh subsets cell percentage among Tfh cells in patients with MM during newly diagnosed, non-remission, and remission states. **(B)** PD-1^+^ICOS^-^ Tfh1 subsets cell percentage among Tfh1 cells in patients with MM during newly diagnosed, non-remission, and remission states. **(C)** PD-1^+^ICOS^-^ Tfh2 subsets cell percentage among Tfh2 cells in patients with MM during newly diagnosed, non-remission, and remission states. **(D)** PD-1^+^ICOS^-^ Tfh17 subsets cell percentage among Tfh17 cells in patients with MM during newly diagnosed, non-remission, and remission states. **(E)** PD-1^-^ICOS^+^ Tfh subsets cell percentage among Tfh cells in patients with MM during newly diagnosed, non-remission, and remission states. **(F)** PD-1^-^ICOS^+^ Tfh1 subsets cell percentage among Tfh1 cells in patients with MM during newly diagnosed, non-remission, and remission states. **(G)** PD-1^-^ICOS^+^ Tfh2 subsets cell percentage among Tfh2 cells in patients with MM during newly diagnosed, non-remission, and remission states. **(H)** PD-1^-^ICOS^+^ Tfh17 subsets cell percentage among Tfh17 cells in patients with MM during newly diagnosed, non-remission, and remission states. “*”, “**”, “***” represent P<0.05, P<0.01, P<0.001, respectively.

We further assessed alterations in the PD-1^+^ICOS^-^ Tfh, PD-1^+^ICOS^+^ Tfh, and PD-1^-^ICOS^+^ Tfh subsets; interestingly, PD-1^+^ICOS^-^ Tfh subsets (including PD-1^+^ICOS^-^ Tfh1, PD-1^+^ICOS^-^ Tfh2, and PD-1^+^ICOS^-^ Tfh17) all showed a gradual decrease from the newly diagnosed group to the remission group, and no differences were observed between the newly diagnosed group and HCs (**Figures 5A–D**). In contrast, the PD-1^-^ICOS^+^ Tfh subsets (including PD-1^-^ICOS^+^ Tfh1, PD-1^-^ICOS^+^ Tfh2, and PD-1^-^ICOS^+^ Tfh17), particularly PD-1^-^ICOS^+^ Tfh2 (P = 0.0005), showed a gradual increase from the newly diagnosed group to remission group (**Figures 5E–H**).

### 3.3 Percentage of Th cell subsets in the BM of patients with MM during different states

**Figure 6:**
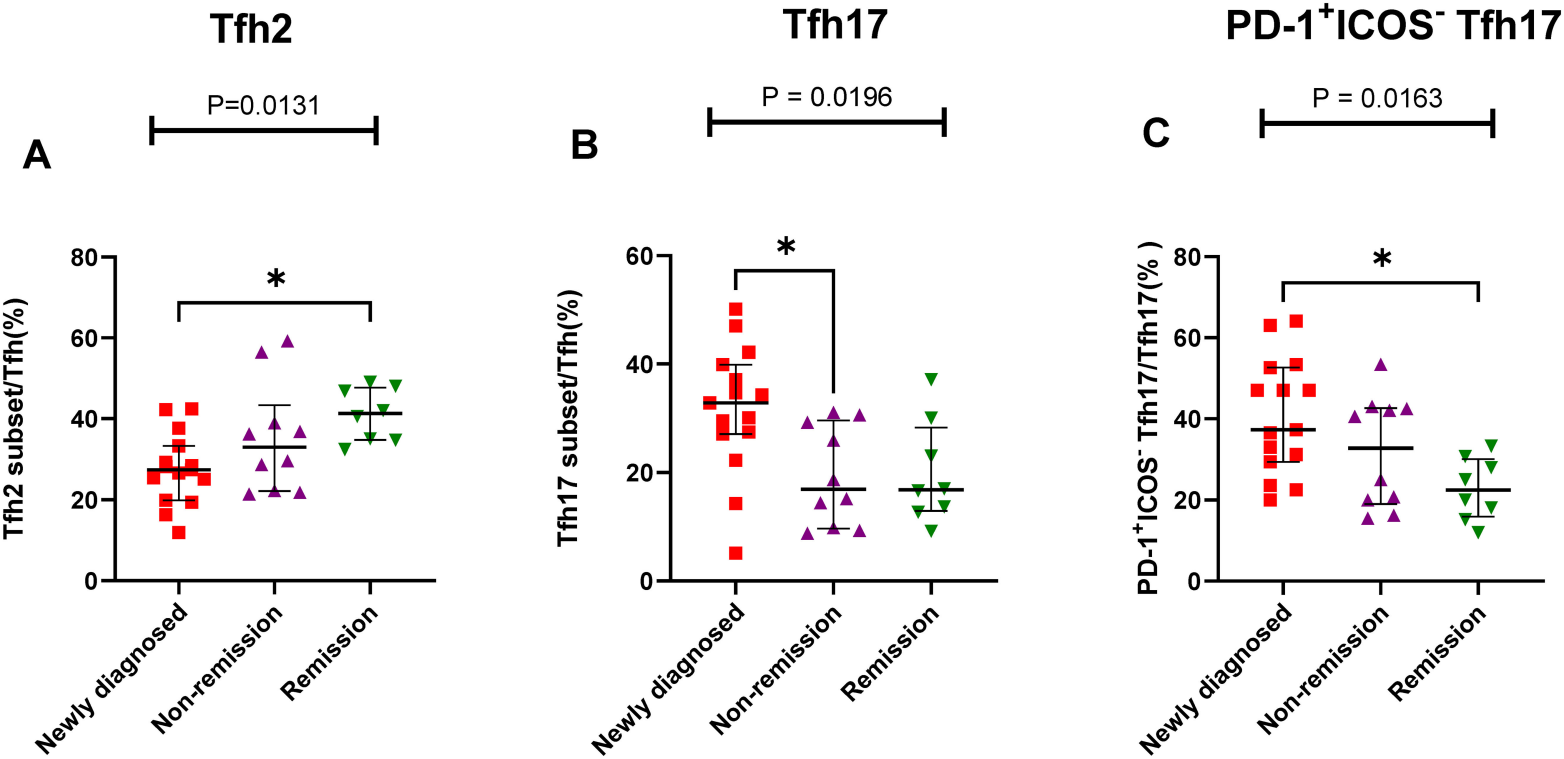
Th cell subsets in the BM samples of patients with MM during different states. **(A)** Tfh2 subsets cell percentage among Th cells in patients with MM during newly diagnosed, non-remission, and remission states. **(B)** Tfh17 subsets cell percentage among Tfh cells in patients with MM during newly diagnosed, non-remission, and remission states. **(C)** PD-1^+^ICOS^-^ Tfh17 subsets cell percentage among Tfh17 cells in patients with MM during newly diagnosed, non-remission, and remission states. “*”, represent P<0.05.

The results showed that the proportion of Th cell subsets varied greatly among the different states in patients with MM. The percentage of Tfh2/Tfh cells gradually increased from the newly diagnosed to non-remission and remission groups, and a significant difference was observed between the newly diagnosed and remission groups (P = 0.105) (**Figure 6A**). However, The percentage of Tfh17/Tfh cells showed a gradual downward trend from the newly diagnosed group to the remission group, there were significant differences among the three groups (P = 0.196) (**Figure 6B**). Among the PD-1^+^ICOS^-^ Tfh subset, the percentages of PD-1^+^ICOS^-^ Tfh17 in the newly diagnosed group were significantly higher than those in the remission group (P = 0.0133) (**Figure 6C**).

### 3.4 Diagnostic value of the proportion of Th subsets in MM

**Figure 7:**
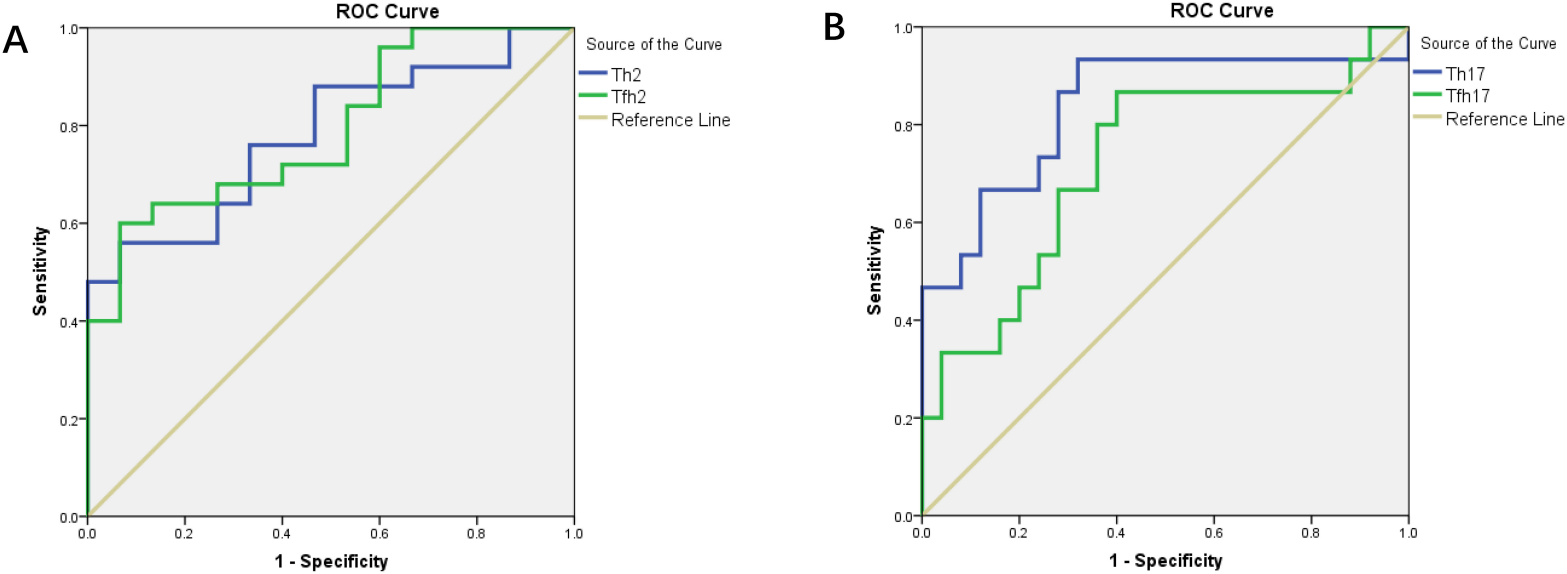
**(A)** ROC curves of Th2 and Tfh2 cells for MM diagnosis. **(B)** ROC curves of Th17 and Tfh17 cells for MM diagnosis.

We found that the percentages of Th subsets in the PB samples of 15 patients with newly diagnosed MM were significantly different from those in HCs (P < 0.05). Therefore, we analyzed the ROC curve to determine the diagnostic effect of the four types of cells (Th2, Th17, Tfh2, and Tfh17) (**Figure 7**). The values for area under the curve (AUC) for percentages of Th2, Th17, Tfh2, and Tfh17 cells were 0.781 (95% confidence interval [CI]: 0.641–0.922), 0.837 (95% CI: 0.694–0.981), 0.792 (95% CI: 0.653–0.931), and 0.723 (95% CI: 0.552–0.894); the sensitivity of diagnosing MM was 56.0, 93.3, 60.0, and 86.7%; and the specificity was 99.4, 68.0, 99.3, and 60.0%, respectively. These data indicated that the percentages of these cells had a good prediction value, with the best for Th17 (**Table 2**).

**Table 2:** Diagnostic value of Th2, Th17, Tfh2, Tfh17 cells in MM.

index	AUC	P value	95% CI	Sensitivity (%)	Specificity (%)	Cut-off value (%)
Th2	0.781	0.003	0.641–0.922	56.0	99.4	63.90
Th17	0.837	<0.001	0.694–0.981	93.3	68.0	9.88
Tfh2	0.792	0.002	0.653–0.931	60.0	99.3	36.60
Tfh17	0.723	0.020	0.552–0.894	86.7	60.0	30.59

### 3.5 Correlation analysis of the percentage of Th subsets between the PB and BM samples of patients with MM

**Figure 8:**
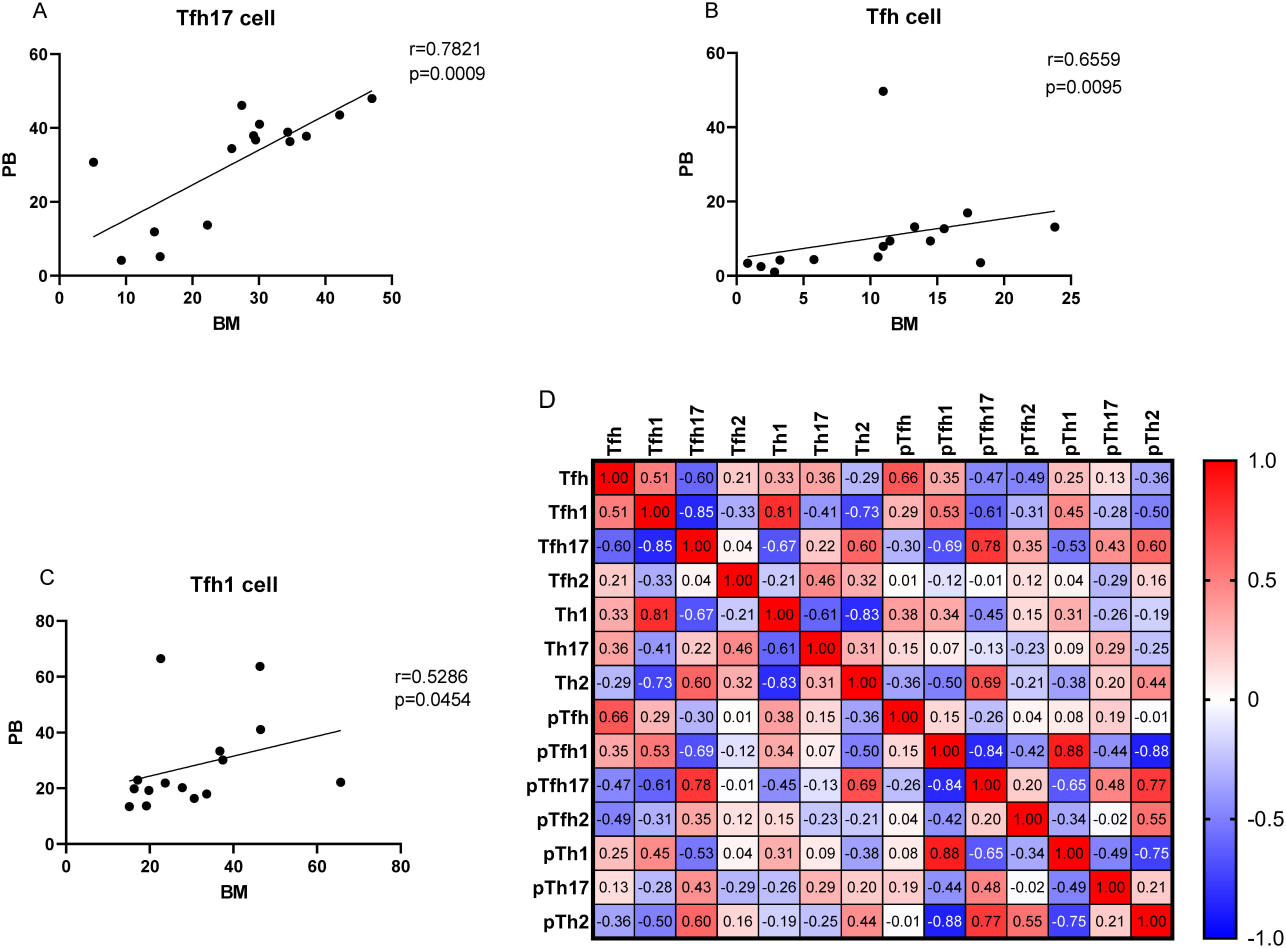
**(A–C)** Correlation between the percentages of Tfh, Tfh1, and Tfh17 cells in the PB and BM samples from patients with MM patients. **(D)** Heat map of the correlation values between all Th cell subsets in the BM and PB samples. Red and blue colors represent positive and negative correlations, respectively.

As no differences were observed in the percentages of Th cell subsets between the PB and BM samples of patients with MM (P > 0.05), we conducted a correlation analysis. The percentage of Tfh17/Tfh cells showed a strong correlation between the BM and PB samples (r = 0.7821, P = 0.0009) (**Figure 8A**). The percentages of Tfh/CD4^+^ and Tfh1/Tfh cells were also correlated (r = 0.6559, P = 0.0095; r = 0.5286, P = 0.0454, respectively) (**Figures 8B, C**).

**Figure 9:**
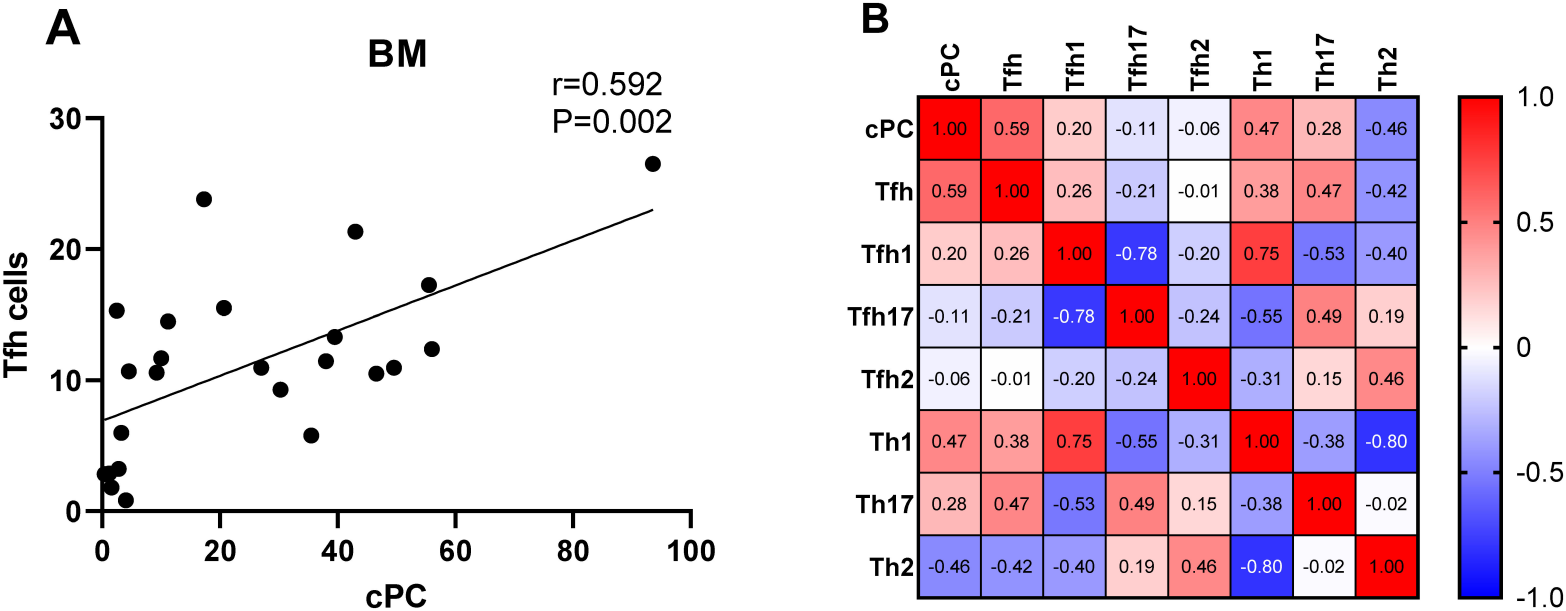
**(A)**. Correlation between the percentage of cPCs and Tfh cell subsets in the BM samples. **(B)**. Heat map of the correlation values between the percentage of cPCs and Th cell subsets in the BM samples. Red and blue colors represent positive and negative correlations, respectively.

**Figure 10:**
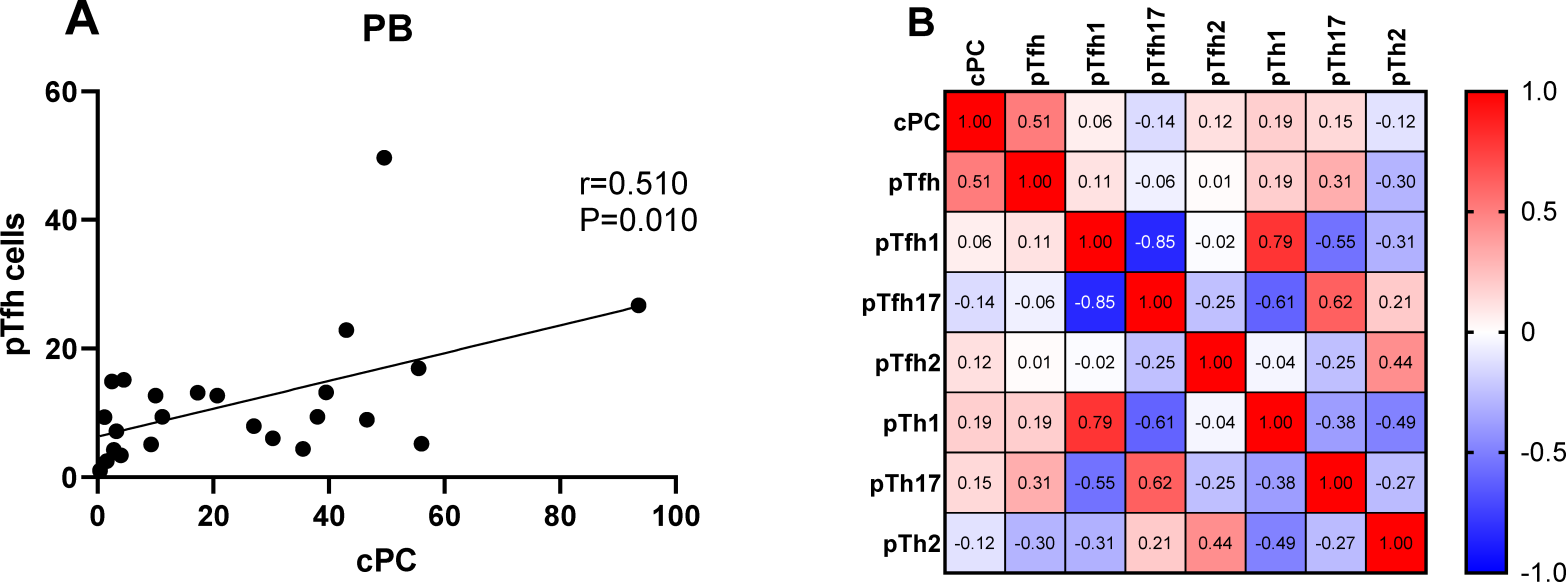
**(A)** Correlation between the percentages of cPCs and Th cell subsets in the PB samples. **(B)** Heat map of the correlation values between the percentage of cPCs and Th cell subsets in the PB samples. Red and blue colors represent positive and negative correlations, respectively.

We determined the percentage of clonal PCs (cPCs) in BM samples. Further, we performed a correlation analysis between the percentages of cPCs and Th cell subsets in the BM and PB samples. The percentage of cPCs had a strong positive correlation with Tfh/CD4^+^ cells in the BM samples (r = 0.592, P = 0.002) (**Figure 9**). In addition, the percentage of cPCs also had a positive correlation with Tfh/CD4^+^ cells in the PB samples (r = 0.510, P = 0.010) (**Figure 10**).

## 4 Discussion

Th cells are the main helper T cells that assist B cells in differentiation and maturation. Th cell subsets are closely associated with the disorder of the immune microenvironment, and the disorder of Th cell subsets also affects the hematopoietic microenvironment, resulting in the occurrence and development of a variety of blood diseases (17). Tfh cells mainly contribute to the activation and proliferation of B cells, production of high-affinity antibodies, transformation of immunoglobulin types, and formation of the germinal center (GC) (18, 19). MM is a hematological malignant tumor characterized by the proliferation of cPCs; however, the role of Th cell subsets in the proliferation and differentiation of cPCs in the PB and BM remains unclear. Therefore, in this study, we explored the changes in the distribution of Th cell subsets in the PB and BM of patients with MM.

The traditional idea is that CD4^+^ T cells play a very important role in B cell activation and humoral immunity, but how T cells assist the germinal center or regulate B cells has been puzzled. With the exploration of researchers, Tfh cells are found to be the key cells that assist B cell differentiation in the germinal center. Normal plasma cells are derived from B lymphocytes, plasma cells can secrete specific immunoglobulins and participate in specific humoral immunity, this process is a key step in whether the body can produce acquired humoral immunity. It is also an important target for clinical treatment of autoimmune diseases and MM (20). CXCR5 molecules recruits Tfh cells to lymphoid follicles for interacting with B cells. Tfh cells can interact with CD40 and ICOSL on B cells through CD40L and ICOS to enhance the activation of B cells and stimulate the proliferation and differentiation of B cells (21). After antigen stimulation, most of the B cells in the GC region will be transformed into plasma cells (22). Current studies have shown that GC, follicular B cells and plasma cells cannot be formed after blocking Tfh cell differentiation and development (23). At present, Tfh cells significantly altered in B-cell lymphoma disease, and are closely related to the prognosis of the disease (24, 25). Our results showed that the proportion of Tfh cells in the PB and BM was positively correlated with that of the myeloma cells, and showed a strong correlation between the BM and PB samples, indicating that Tfh cells may be biomarkers reflecting myeloma cells in BM and PB. However, the sample size is relatively low and it needs to be further confirmed by further expansion of cases and *in vitro* experiments.

Other iconic molecules, such as ICOS and PD-1, on the surface of Tfh cells play important roles in their migration, localization, differentiation, and development. The abnormal expression of these molecules may result in a change in Tfh function (26, 27). ICOS, a marker of T cell activation, can bind to the CD278 ligand on B cells to transmit stimulatory signals, which play an important role in promoting the expression of interleukin 21 (IL-21) by Tfh cells and assist in the formation of memory B cells (28). PD1 is an immunosuppressor receptor that inhibits T lymphocyte proliferation, produces cytokines, and weakens ICOS and IL-21 activation in Tfh cells (29). Thus, PD-1 transmits negative signals. Increased levels of ICOS^+^ and PD-1^+^ T cells have been observed in patients with rheumatoid arthritis and systemic lupus erythematosus (30–32). In our studies, all subsets of PD-1^+^ICOS^-^ Tfh (PD-1^+^ICOS^-^ Tfh1, PD-1^+^ICOS^-^ Tfh2, and PD-1^+^ICOS^-^ Tfh17) in patients MM patients gradually decreased after chemotherapy in the PB samples. Notably, in the BM samples, the percentage of PD-1^+^ICOS^-^ Tfh17 cells subsets in the newly diagnosed patients with MM was significantly higher than that in remission patients with MM. Some studies have shown that the upregulated PD-1 expression on Tfh myeloma cells may block the activation signal of T cells in a variety of ways, inhibiting the proliferation of T cells (33), forming a microenvironment of MM immune escape, and promoting MM progression. Overexpression of PD-1 leads to T cell dysfunction in hematological malignancies, such as non-Hodgkin’s tumors, acute myeloid leukemia (AML), and chronic lymphocytic leukemia (34–36). In our study, PD-1 expression in patients with MM gradually decreased after chemotherapy, indicating that the immunosuppressive state gradually weakened and returned to normal after complete remission. In contrast, all subsets of PD-1^-^ICOS^+^ Tfh (PD-1^-^ICOS^+^ Tfh1, PD-1^-^ICOS^+^ Tfh2, and PD-1^-^ICOS^+^Tfh17) in the PB samples of patients with MM showed a gradual increase after chemotherapy. ICOS, a positive stimulatory molecule, is mainly expressed on the surface of activated T lymphocytes, particularly Tfh cells, and promotes their transformation (37). Notably, in ICOS-knockout mice, germinal center formation, B cell maturation, and antibody class transformation are impaired and the expression of Tfh cells is significantly reduced (38). In contrast, in mice with ICOS gene transfer, high ICOS expression promotes the formation of germinal center and differentiation and proliferation of Tfh cells (39). Our study showed that after chemotherapy, ICOS expression was gradually upregulated, indicating that ICOS could gradually promote the proliferation of Tfh cells and recover immune function, which is conducive to the recovery of diseases. However, which needs to be further experimental results expanded to confirm.

Th1 cells mainly participate in the cellular immune response; Th2 cells mainly secrete cytokines, promote the activation of B cells, and mediate the humoral immune response (40); and Th17 cells produce pro-inflammatory factors that can trigger inflammatory responses and play an important role in mediating host defense (41). In our study, the percentages of Th2/Th cells in the PB samples of patients with MM were significantly lower than those in HCs. Further analysis showed that the proportion of Th2 cells in newly diagnosed patients with MM was significantly lower than that in HCs, which gradually decreased after chemotherapy and stabilized after complete remission in patients with MM. Notably, Th17 cells showed the opposite result, the percentage of Th17/Th cells in the PB samples of patients with MM was significantly higher than that in HCs and the same as that in the newly diagnosed patients with MM. Similar results have been reported in recent years for immune-related blood diseases, such as aplastic anemia (AA) and autoimmune hemolytic anemia (AIHA), studies have reported a decrease and increase in the percentages of Th2 and Th17 cells, respectively (42). they indicated that Th2 and Th17 cells are essential for maintaining humoral immune homeostasis, and imbalances may lead to the development of disease (43, 44). Th subsets in the PB showed a high diagnostic value for MM, and the diagnostic efficiency of Th2 and Th17 were high. In particular, Th17 cells showed the highest diagnostic efficiency, with 93.3 and 68% sensitivity and specificity, respectively, which is of great significance for the early diagnosis of MM and could be used as a marker in MM for early disease screening.

Different from Th subsets, the characteristic surface markers of Tfh subsets were CXCR5, PD-1 and ICOS. Tfh subgroup cells are actually the main helper T cells that play an active role in the differentiation of B cells into PCs (45). Although Tfhl cells lack the ability to induce B cells to produce antibodies, Tfh2 cells promote IgG and IgE secretion, and Tfh17 cells induce IgG and IgA secretion (12). In our study, Th and Tfh subgroup have similar results, the percentage of Tfh2/Tfh cells in the PB samples of patients with MM was significantly lower than that in HCs, after chemotherapy, the proportion of Tfh2 cells in remission group with MM increased to normal. Similar results also appeared in the BM samples, from newly diagnosis to complete remission groups, the proportion of Tfh2 in the patients with MM gradually increased. In contrast, the percentage of Tfh17/Th cells in patients newly diagnosed with MM was higher than that in HCs, and Tfh17 cells in remission group with MM decreased to normal in the PB and BM sample. Ye et al. (15) also reported a significantly higher number of Tfh17 cells in the PB of patients with MM than that in HCs, whereas the number of Tfh2 cells was significantly lower than that in HCs. Tfh17 cells directly increase disease progression and promote cell growth in MM (46, 47). Tfh2 and Tfh17 cells may play important roles in the growth and differentiation of normal and malignant PCs in patients with MM. In terms of the relationship between the proportion of Tfh cell subsets in the PB and BM, we found that the percentages of Tfh17 cells in the BM were best correlated with those in the PB. Tfh17 cells are characterized by the CCR6, which is the receptor for the chemokine CCL20. It has been reported that in a variety of cancers, CCR6^+^ tumor cells can migrate to CCL20 enrichment sites and promote tumor metastasis (48, 49). Myeloma cells are widely distributed in both PB and BM, so Tfh17 cells may better reflect the development of tumor cells in bone marrow than Tfh2 cells, which needs to be further expanded to confirm. This finding is of great significance for clinical studies. When patients are tested for cellular immune function, the proportion of Tfh17 cells in the PB may better represent their proportion in the BM, and the extraction of BM can be avoided, reducing patient suffering and medical expense.

## 5 Conclusion

The proportion of Tfh cells positively correlated with that of myeloma cells in the BM and PB. and showed a strong correlation between the BM and PB samples, indicating that Tfh cells may be biomarkers reflecting myeloma cells in BM and PB. In the PB, PD-1 expression gradually decreased, whereas ICOS expression gradually increased from the newly diagnosed to the non-remission groups and the remission group after chemotherapy. These findings suggest that PD-1 and ICOS may be closely related to chemotherapy. The proportion of Th2 and Tfh2 cells in the PB of newly diagnosed patients with MM was significantly lower than in HCs, whereas the proportion of Th17 and Tfh17 cells was higher than in HCs. All these cells showed good diagnostic value and can be used for the early diagnosis of MM. Therefore, Th cell subsets play an important role in the occurrence and development of MM.

## Data Availability

The original contributions presented in the study are included in the article/supplementary material. Further inquiries can be directed to the corresponding author.
